# Interleukin 15 (IL15)-based near-infrared photoimmunotherapy

**DOI:** 10.1007/s00262-025-04151-8

**Published:** 2025-08-31

**Authors:** Motofumi Suzuki, Aki Furusawa, Hiroshi Yamamoto, Makoto Kano, Miyu Kano, Seiichiro Takao, Shuhei Okuyama, Peter L. Choyke, Hisataka Kobayashi

**Affiliations:** Molecular Imaging Branch, Center for Cancer Research, National Cancer Institute, National Institutes of Health, 10 Center Drive, Bethesda, MD 20892 USA

**Keywords:** Interleukin-15, Immunogenic cell death, Near-infrared photoimmunotherapy, Tumor treatment

## Abstract

Near-infrared photoimmunotherapy (NIR-PIT) is a highly selective cancer therapy based on a targeted monoclonal antibody conjugated to phthalocyanine-based photoabsorber, IRDye700DX (IR700). Following injection, near-infrared light activates the conjugate causing direct cytotoxicity and immunogenic cell death. The high selectivity of NIR-PIT is traditionally based on the binding of a monoclonal antibody to its target thus bringing the photoabsorber, IR700, in close proximity to the cell membrane at the time of light exposure. However, other targeting moieties can be used and in the current study, interleukin-15 (IL15) is conjugated with IR700 to enable NIR-PIT. IL15-IR700 quickly bound to the cellular membrane of MC38 HIL15Rα cells that are transfected with human IL15 receptor alpha. NIR-PIT induced characteristic morphological changes accompanied by a significant decrease of cellular viability. Intratumoral injection of IL15-IR700 resulted in longer retention of the conjugate in the tumor compared to the intravenous injection. NIR-PIT with intratumoral IL15-IR700 significantly suppressed tumor growth, induced necrosis-like changes in tumor tissue, and stimulated host immune response. This study revealed that cytokine-based NIR-PIT is a potential therapeutic strategy for treating solid cancer.

**Supplementary Information:**

The online version contains supplementary material available at 10.1007/s00262-025-04151-8.

## Introduction

Near-infrared photoimmunotherapy (NIR-PIT) is a newly developed, highly selective cancer treatment. NIR-PIT is based on the injection of a conjugate consisting of a monoclonal antibody and a phthalocyanine-based photoabsorber, IRDye700DX (IR700), which absorbs light at 690 nm in the NIR range [1, 2]. Exposure to NIR-light induces a photochemical reaction that disrupts membrane integrity causing cell death [3]. Cellular membrane rupture causes the release of cellular contents which stimulates vigorous host immune response leading to immunogenic cell death (ICD) [2]. NIR-PIT targeting various membrane proteins, including epidermal growth factor receptor (EGFR) and human EGFR2 among many others, have been developed and exhibit therapeutic efficacy in preclinical models [4]. A global phase III randomized controlled trial of NIR-PIT utilizing anti-EGFR-IR700 in patients with recurrent head and neck carcinoma is currently ongoing (NCT03769506). Moreover, in Japan, NIR-PIT with Cetuximab-IR700 (Akalux™; Rakuten Medical Inc., San Diego, CA, USA) was approved for clinical use in September 2020.

The high selectivity of NIR-PIT is ensured by a combination of the tumor-targeting monoclonal antibody and the focal application of NIR-light. While antibodies are highly effective for NIR-PIT, there are several limitations including complexity and cost of producing medical grade antibodies. In addition, due to their large molecular weight, antibodies often have limited tumor penetration reducing coverage [5]. Thus, we sought to identify alternative targeting agents for NIR-PIT [6]. Although there have been attempts to utilize molecules other than antibodies, these efforts have not been successful in vivo [7–9].

In this study, we aimed to test the feasibility of utilizing cytokines as NIR-PIT agents. Cytokines are soluble proteins with low molecular weight (6–70 kDa) that are secreted by a variety of cells [10]. Cytokines cannot cross the lipid bilayer due to their size; thus, cytokines bind to cell surface receptors thereby triggering an intracellular signal cascade. Most cytokine-based cancer therapies take advantage of a direct anti-proliferative effect or function more indirectly by stimulating anti-tumor immunity [11]. It has been previously shown that intratumoral injection of interleukin-15 (IL15) potentiated the therapeutic effects of NIR-PIT by enhancement of T cell response in syngeneic mouse tumors [12, 13]. However, cytokine-based NIR-PIT has not yet been developed. In the current study, we evaluate the effectiveness of IL15-based NIR-PIT both in vitro and in vivo and assess the host immune response after this therapy.

## Material and methods

### Reagents

A water-soluble, silicon-phthalocyanine derivative, IRDye700DX N-hydroxysuccinimide ester (IR700-NHS) was purchased from Li-COR Bioscience (Lincoln, NE). IL15 was obtained from BRB Preclinical Biologics Repository (Frederick, MD). All other chemicals were of reagent grade.

### Synthesis of IR700-conjugated IL15

IL15 (160 μg, 0.12 nmol) was incubated with IR700-NHS (120 μg, 0.62 nmol) in 0.1 M Na_2_HPO_4_ (pH 8.5) at room temperature for 1 h. Upon the removal of both the lower and upper caps from a Sephadex G25 column (PD-10; Cytiva, Marlborough, MA), the process of equilibration was executed by circulating PBS through the column three times. Following the addition of 300 μl of the mixture, which was permitted to incubate for one minute, an additional volume of 2400 μl of PBS was introduced, and subsequently, 1800 μl of the resultant mixture was collected to obtain IR700-conjugated IL15 (IL15-IR700). The protein and IR700 concentration were determined by previously described methods [14, 15]. The synthesis was controlled so that an average of two IR700 molecules were bound to a single IL15.

### Cell culture

Murine colon cancer MC38 cells transfected with human IL15 receptor alpha (MC38 HIL15Rα) were used in this study [16]. MC38 was kindly provided by Dr. Thomas Waldmann (National Institute of Health, Bethesda, MD). Human breast cancer cell line MDA-MB-231 was obtained from the National Cancer Institute Frederick Cancer Division of Cancer Treatment and Diagnosis Tumor/Cell Line Repository (Frederick, MD). All cell lines were maintained in RPMI1640 medium (Thermo Fisher Scientific, Rockford, IL) supplemented with 10% (v/v) fetal bovine serum (FBS; Thermo Fisher Scientific) and antibiotics (100 μg/mL penicillin and streptomycin, Thermo Fisher Scientific) at 37 °C in a humidified atmosphere of 5% CO_2_.

### Flow cytometry

For binding assay, cells were collected and washed with ice-cold staining buffer (SB: PBS supplemented with 1% FBS). One million cells were incubated in the dark with IL15-IR700 (0.5 μg) for 10 min at 4 °C. Following two washings with cold-SB, the cells were analyzed using BD FACSLyric flow cytometer (BD Biosciences, San Jose, CA) and FlowJo software (FlowJo LLC, Ashland, OR). For blocking assay, the cells were pre-incubated with a 100-fold molar excess of unconjugated IL15 for 15 min followed by incubation with IL15-IR700.

For ICD marker detection, the cells were collected immediately after NIR-PIT and incubated in the dark with phycoerythrin-conjugated anti-heatshock protein 70 (HSP70; clone REA349; Miltenyi Biotec, Gaithersburg, MD) and anti-calreticulin (rabbit poly; Bioss Antibodies, Woburn, MA) for 30 min at 4 °C.

For phenotypic analysis of immune cells in tumor draining lymph node (TDLN), inguinal lymph nodes from tumor-bearing mice were excised as TDLNs two days after NIR-PIT. Lymphoid cells were isolated by mechanical disruption of TDLN, and cell suspension was filtered through a 40 μm cell strainer and washed with cold-SB. Surface staining was performed using antibodies for the following markers: CD3e (clone 145–2C11; Biolegend, San Diego, CA), CD4 (clone RM4-5; Biolegend), CD8 (clone 53-6.7; Thermo Fisher Scientific), CD25 (clone PC61; Biolegend), CD45 (clone 30-F11; Biolegend), CD69 (clone H1.2F3; Biolegend), and NK1.1 (clone PK136; Biolegend). All cells were then stained with eBioscience™ Fixable Viability Dye eFluor™ 780 (Thermo Fisher Scientific).

### Fluorescence microscopy

Cells were seeded onto a glass bottom dish and cultured overnight. Then, the cells were exposed to phenol-red free media containing IL15-IR700 (0.5 μg/mL) for 10 min at 37 °C. Fluorescent microscopic analysis was performed using an Olympus IX81 microscope (Olympus America, Melville, NY) with a reflected light fluorescence.

### Cell viability assay

Cell viability assay was performed as previously described [14, 15]. Briefly, the cells were exposed to IL15-IR700 (0.5 μg/mL) for 10 min at 37ºC and irradiated with NIR-light. After incubation for 3 h, cell viability was evaluated by MTT assay. For the blocking assay, the cells were pre-treated with unconjugated IL15 (5 μg/mL) for 10 min at 37 °C. After washing with PBS, the cells were treated with IL15-IR700 (0.5 μg/mL) for 10 min at 37 °C and irradiated.

### Animal experiments

All animal experiments were performed according to the established guidelines of the “Guide for the Care and Use of Laboratory Animals” and approved by the Institutional Animal Care and Use Committee (MIP-003-4-AA). All animal experiments were conducted with a minimum of three mice. Five mice per cage were housed in individual cages with enrichment in temperature-controlled rooms and had free access to water and food. Two million MC38 HIL15Rα cells were injected subcutaneously into 7-week-old female C57BL/6J mice (strain #000664; The Jackson Laboratory, Bar Harbor, ME). When the tumor volume exceeded 50 mm^3^, the mice were randomly divided into three groups using TumorManager software (Biopticon, Princeton, NJ): (i) no treatment; (ii) intertumoral injection of IL15-IR700 (5 μg) without NIR-light exposure; (iii) intratumoral injection of IL15-IR700 (5 μg) with NIR-light exposure (50 J/cm^2^ at 5 h after IL15-IR700 injection). The dosage for intratumoral administration was based on previous report [13]. NIR-light exposure was performed using the ML7710 laser system (Modulight, Tampere, Finland). IR700 fluorescence imaging before and immediately after light exposure was conducted using a Pearl Imager (LI-COR Bioscience). Tumor diameter and body weight were monitored every 2 days. The tumor volume was calculated using the following formula: tumor volume (mm^3^) = length (mm) × width (mm) × width (mm) × 0.5. Mice were euthanized with inhalation of carbon dioxide gas when tumor volume reached 2000 mm^3^.

### Distribution analysis of digoxigenin-conjugated IL15

IL15 (160 μg, 0.12 nmol) was incubated with digoxigenin (DIG) NHS-ester (8 μg) in 0.1 M Na_2_HPO_4_ (pH 8.5) at room temperature for 1 h. The mixture was purified with a Sephadex G25 column (PD-10) to obtain DIG-conjugated IL15 (IL15-DIG). Five hours after intratumoral injection of IL15-DIG (5 μg), the tumor xenograft was excised and fixed with 10% formalin solution (Sigma-Aldrich). The distribution of IL15-DIG was assessed by immunohistochemistry.

### Immunohistochemistry and histological analysis

Immunohistochemistry analysis was performed as previously described [14, 15]. The following antibodies were used: anti-DIG (clone 9H27L19; Thermo Fisher Scientific), anti-Ki67 (clone D3B5; Cell Signaling Technology, Danvers, MA), and anti-pan Cytokeratin (pCK) (bs-1712R; Bioss Antibodies). All antibodies were diluted to 1:1000. For histological analysis, paraffin sections were stained with hematoxylin and eosin (HE).

#### Statistical analysis

Statistical analysis was performed using GraphPad Prism 10 (GraphPad Software, Inc. Boston, MA). Comparisons of two groups were performed using Student’s *t*-test. For multiple comparisons, the Tukey–Kramer test was used. For comparison of survival, long-rank test was used. The minimum level of significance was set at *P* < 0.05.

## Results

### IL15-IR700 selectively bound to cells

**Fig. 1:**
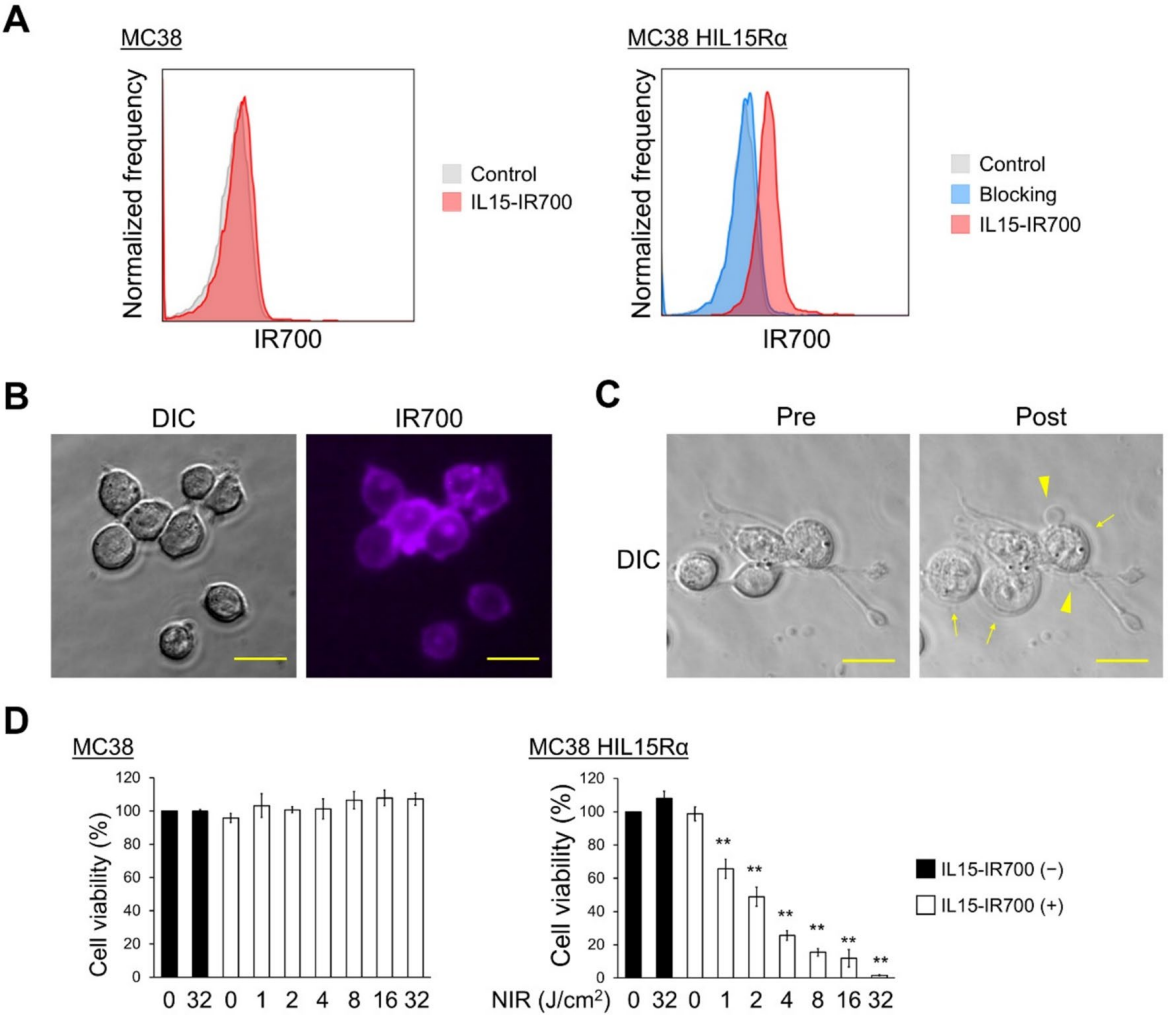
Cytotoxic effects of NIR-PIT. **A** The surface binding of IL15-IR700 on MC38 and MC38 HIL15Rα cells was detected by flow cytometry. The gray, blue, and red histograms represent control, pre-treated with excess IL15 for blocking, and IL15-IR700–bound cells, respectively. **B** Immunofluorescence images of IL15-IR700–bound cells. Scale bar, 20 μm. DIC, differential interference contrast. **C** Representative images of morphological changes, including bleb formation (arrowhead) and swelling (arrow) after NIR-PIT. Scale bar, 20 μm. Pre, before irradiation; Post, after irradiation. **D** Cell viability after NIR-PIT in MC38 and MC38 HIL15Rα cells was evaluated by MTT assay. Data are expressed as the mean ± SD of three independent experiments. **P < 0.01 versus Control (one-way ANOVA followed by Dunnett’s test)

As shown in Supplemental Fig. 1, although the molecular weights of IL15 and IL15-IR700 are almost same, only IL15-IR700 has fluorescence, suggesting that IR700 conjugation for IL15 was successful. Flow cytometry confirmed that IL15-IR700 bound to MC38 HIL15Rα and not to MC38 cells, which do not express the HIL15Rα (Fig. 1A). This binding was inhibited by pre-treatment with IL15, suggesting that IL15-IR700 had the ability to selectively bind cell surfaces on MC38 HIL15Rα cells. The binding of IL15-IR700 on the cell surface was also confirmed by fluorescent microscopy imaging (Fig. 1B).

### Cytokine-based NIR-PIT induced selectively cytotoxicity in vitro

Immediately after NIR irradiation under microscopy, swelling and bleb formation were observed in MC38 HIL15Rα cells (Fig. 1C). These morphological changes are consistent with antibody-based NIR-PIT-induced cell death [1]. The cytotoxicity of NIR-PIT was evaluated at 3 h after NIR-light irradiation (Fig. 1D). NIR-light at 32 J/cm^2^ alone and IL15-IR700 alone without NIR-light exposure had no effect on cellular viability. When IL15-IR700 was combined with NIR-light, cellular viability was significantly decreased in a NIR-light dose-dependent manner. To verify the selectivity of NIR-PIT, the cells were pre-treated with cold IL15, followed by NIR-PIT with IL15-IR700. The cytotoxic effects of NIR-PIT were blocked by pre-treatment with IL15 (Supplemental Fig. 2). Moreover, NIR-PIT had no cytotoxic effects in MC38 cells, which do not express HIL15Rα (Fig. 1D). In addition, the cytotoxic effects of cytokine-based NIR-PIT in human cancer were evaluated in human breast cancer MDA-MB-231 cells with reportedly high levels of IL15Rα [17]. After NIR irradiation under microscopy, dramatic morphological changes were observed, and cellular viability was significantly decreased in a NIR-light dose-dependent manner (Supplemental Fig. 3). These results suggested that cytokine-based NIR-PIT using IL15 as the targeting agent was effective and could induce selective cytotoxic effects in a manner similar to antibody-based NIR-PIT.

### Intratumoral injection of IL15-IR700 was superior to intravenous injection in tumor retention

**Fig. 2:**
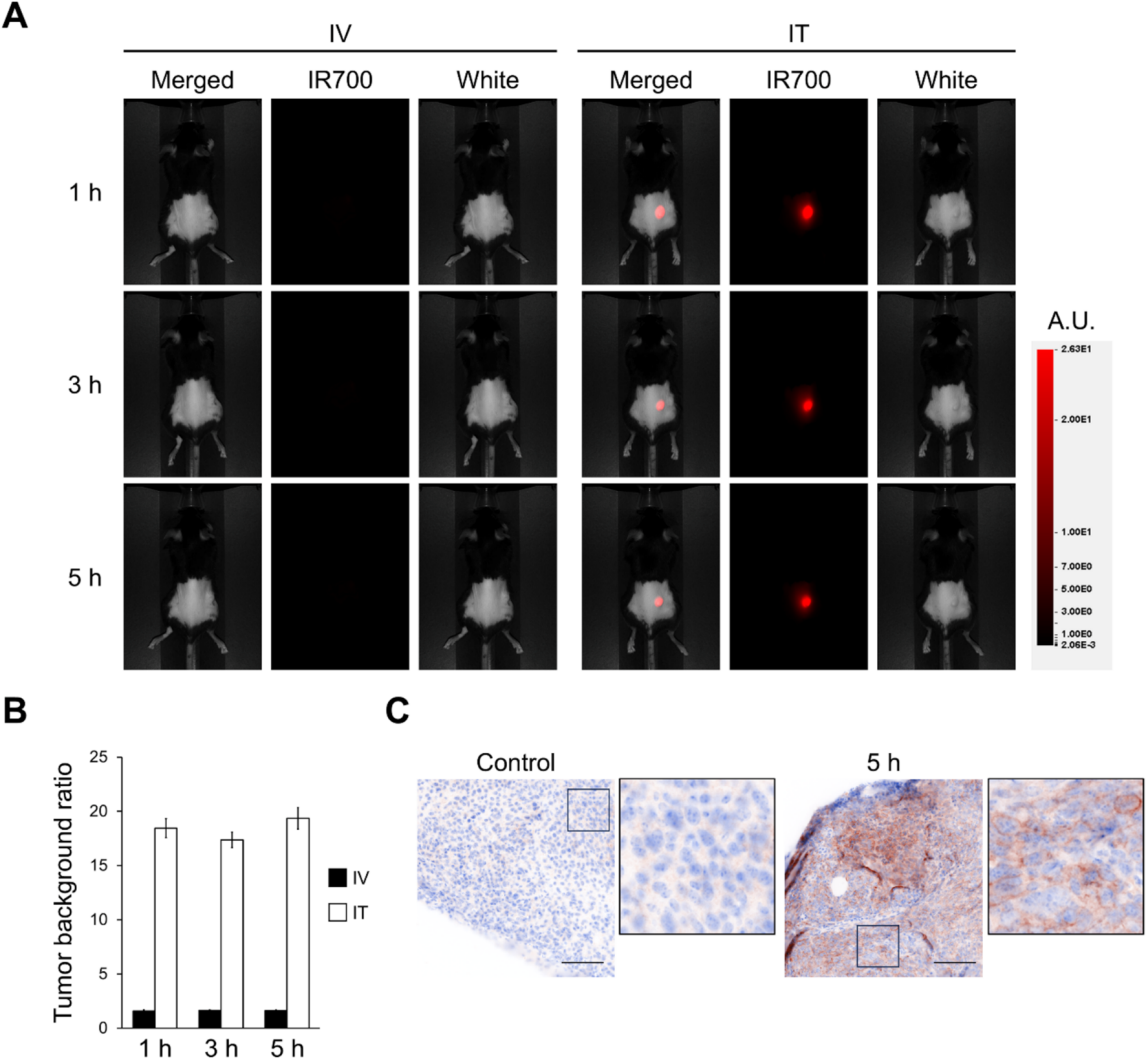
Accumulation of IL15-IR700 in tumor tissue. **A** Fluorescence images taken after intravenous (IV) or intratumoral (IT) injection of IL15-IR700. **B** Quantitative analysis of tumor-background fluorescence intensity ratio in IV and IT injected mice. Data are expressed as the mean ± SD (n = 5). **C** Distribution of IL15 conjugates in tumor tissue. Five hours after intratumoral injection, the distribution was evaluated by immunohistochemistry. Scale bar, 100 μm

In antibody-based NIR-PIT, antibody-IR700 conjugates are intravenously injected into mouse models which accumulate fluorescence as the conjugate binds to the cell; thus, the accumulation of intravenously injected IL15-IR700 in tumors could be assessed by fluorescence imaging. Although the accumulation of IL15-IR700 in tumors was observed, the background signal was high as well (Fig. 2A and Supplemental Fig. 4). To improve the selective IL15-IR700 accumulation in tumors, we used an intratumoral approach. The fluorescence signal was still confined to the tumor even after 5 h post-intratumoral injection (Fig. 2A). The tumor-background ratio in intratumoral injection group was significantly higher than intravenous injection group 5 h after administration (1.62 ± 0.05 and 19.35 ± 0.99, respectively) (Fig. 2B). To evaluate the distribution of IL15 in the tumor, IL15-DIG was intratumorally injected, and the distribution in tumor tissue was detected by anti-DIG immunohistochemistry. As shown in Fig. 2C, DIG was detected on tumor cell surfaces, indicating that intratumorally injected IL15-DIG bound to the cell surface in the tumor. These results suggested that intratumoral injection was more appropriate than intravenous injection in cytokine-based NIR-PIT.

### Cytokine-based NIR-PIT suppressed tumor growth in syngeneic models

**Fig. 3:**
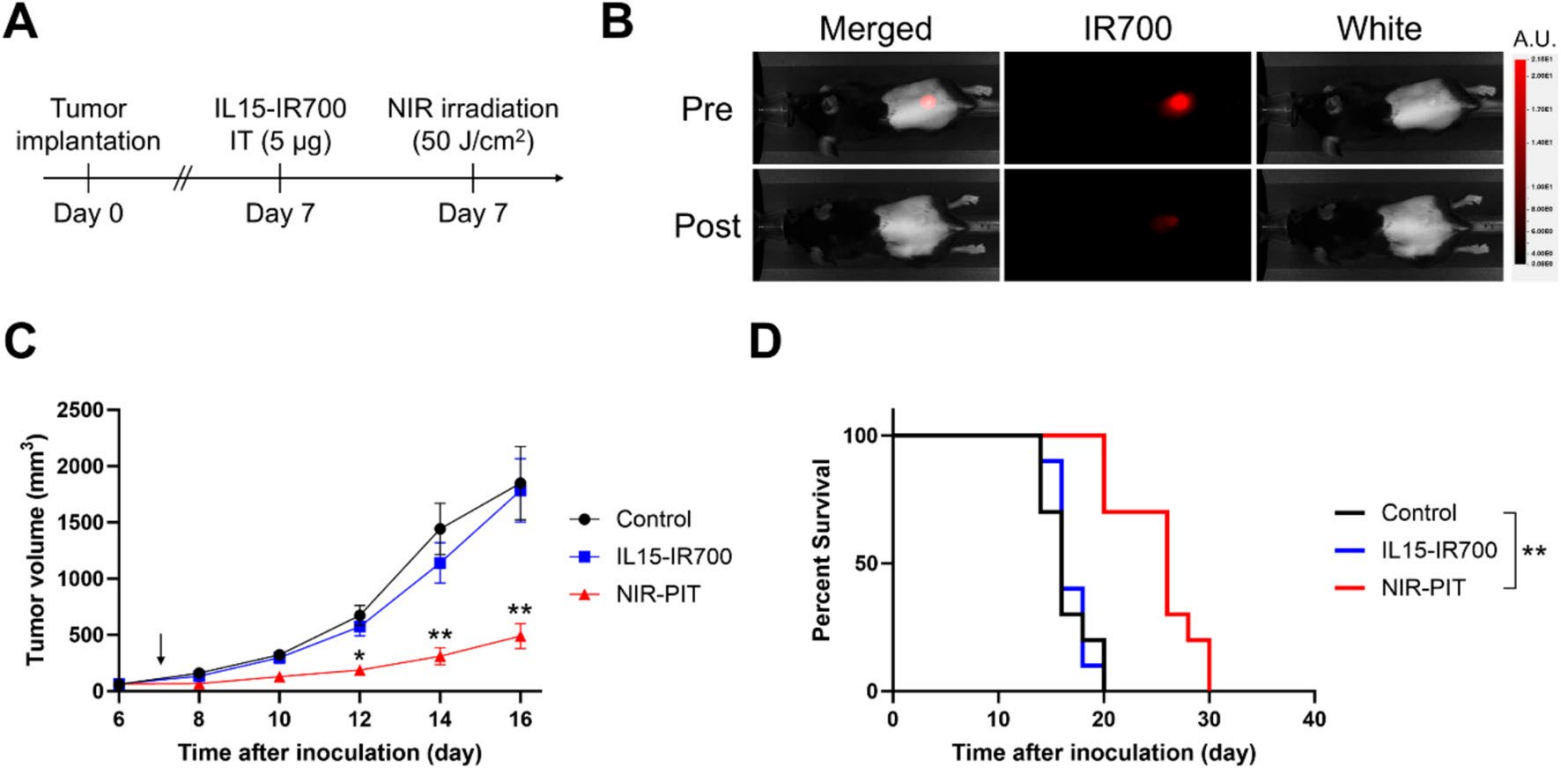
In vivo NIR-PIT in an MC38 HIL15Rα tumor-bearing mouse model. **A** Schematic diagram of IL15-IR700 intratumoral injection and NIR-light exposure. **B** Fluorescence images taken before and immediately after NIR-PIT on Days 7. **C** Tumor growth inhibition by NIR-PIT. The graph presents tumor volumes after treatment. Arrow indicates the day of treatment. Data are expressed as the mean ± SEM. *P < 0.05, **P < 0.01 versus Control (n = 10; Tukey–Kramer test). **D** Survival analysis after treatment. **P < 0.01 versus Control (n = 10; log-rank test with Bonferroni correction)

**Fig. 4:**
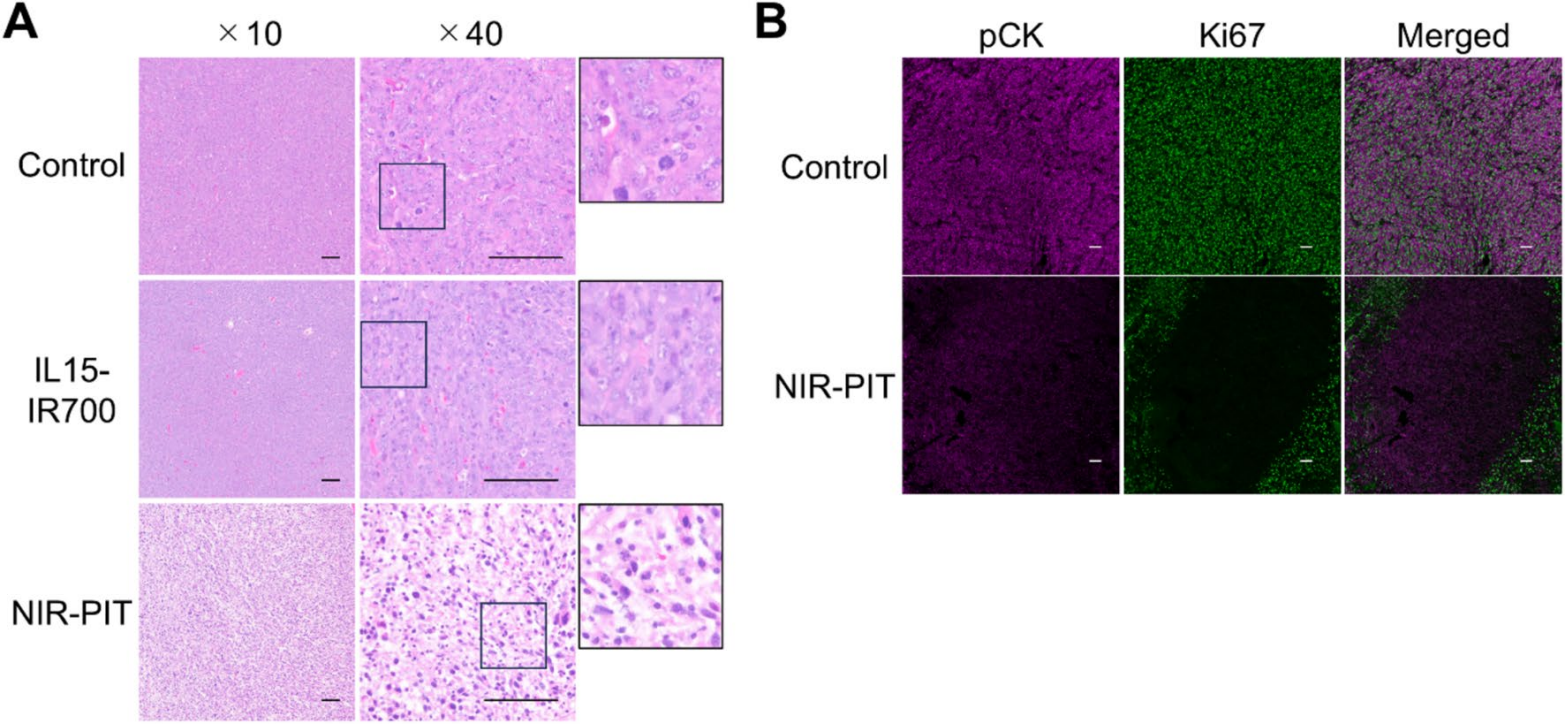
Histological analysis after NIR-PIT. Histological changes were evaluated by HE-staining and immunohistochemistry at 24 h after NIR-PIT. Scale bar, 100 μm. **A** Representative HE-staining of the tumor sections. **B** Representative immunofluorescence images of pan-cytokeratin (pCK; a marker for the differentiation of epithelial and mesothelial cells) and Ki67 (proliferation marker)

The therapeutic effects of NIR-PIT using IL15-IR700 were evaluated in tumor-bearing mice. As shown in Fig. 3A, IL15-IR700 was intratumorally injected in mice 7 days after tumor implantation, and NIR-PIT was performed 5 h after administration of IL15-IR700. The fluorescence signal of IR700 decreased immediately after NIR irradiation (Fig. 3B), suggesting that the expected photochemical reaction had successfully occurred. Tumor growth was significantly inhibited in the NIR-PIT group compared with the control and IL15-IR700-alone group (Fig. 3C). Moreover, NIR-PIT significantly prolonged the survival time of mice compared with the control group (Fig. 3D). In the HE-staining of tumor tissue, NIR-PIT group showed dramatic histological changes, including nuclear pyknosis and eosinophilic cytoplasm, that was not observed in the control and IL15-IR700-alone treatment group (Fig. 4A). In addition, histologic changes were not observed after NIR-PIT with intravenous injection of IL15-IR700 (Supplemental Fig. 5). After 24 h of NIR-PIT, the proliferation marker Ki67 was decreased compared to the control group (Fig. 4B). These results suggested that NIR-PIT using intratumoral IL15-IR700 is effective in vivo.

### Cytokine-based NIR-PIT induced immunogenic cell death in vitro

**Fig. 5:**
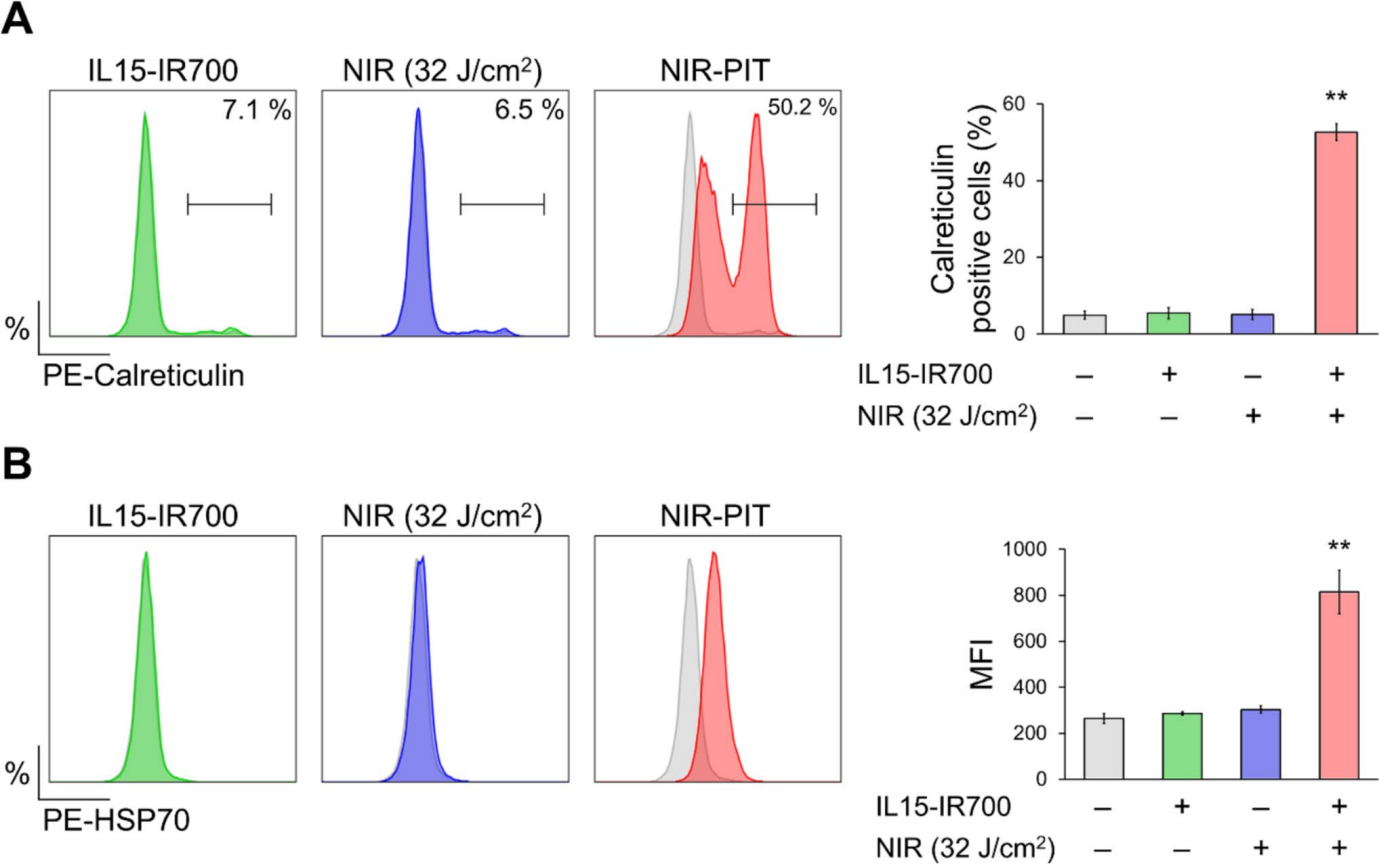
Detection of immunogenic cell death cell surface marker in vitro. Immediately after NIR-PIT, the expression of calreticulin **A** and heatshock protein 70 **B** were evaluated by flow cytometry. Data are expressed as the mean ± SD of three independent experiments. **P < 0.01 versus Control (n = 3, Tukey–Kramer test). MFI; mean fluorescence intensity

Previously, antibody-based NIR-PIT has been reported to induce ICD associated with leakage of cellular contents and stimulation of the host immune system against tumor [3]. The expressions of two ICD-related molecules, HSP70 and calreticulin, were evaluated immediately after in vitro cytokine-based NIR-PIT. As shown in Fig. 5A and B, both ICD-related molecules were significantly increased, suggesting that cytokine-based NIR-PIT also induced ICD and had a potential for immune activation.

### Cytokine-based NIR-PIT activated host immune response

**Fig. 6:**
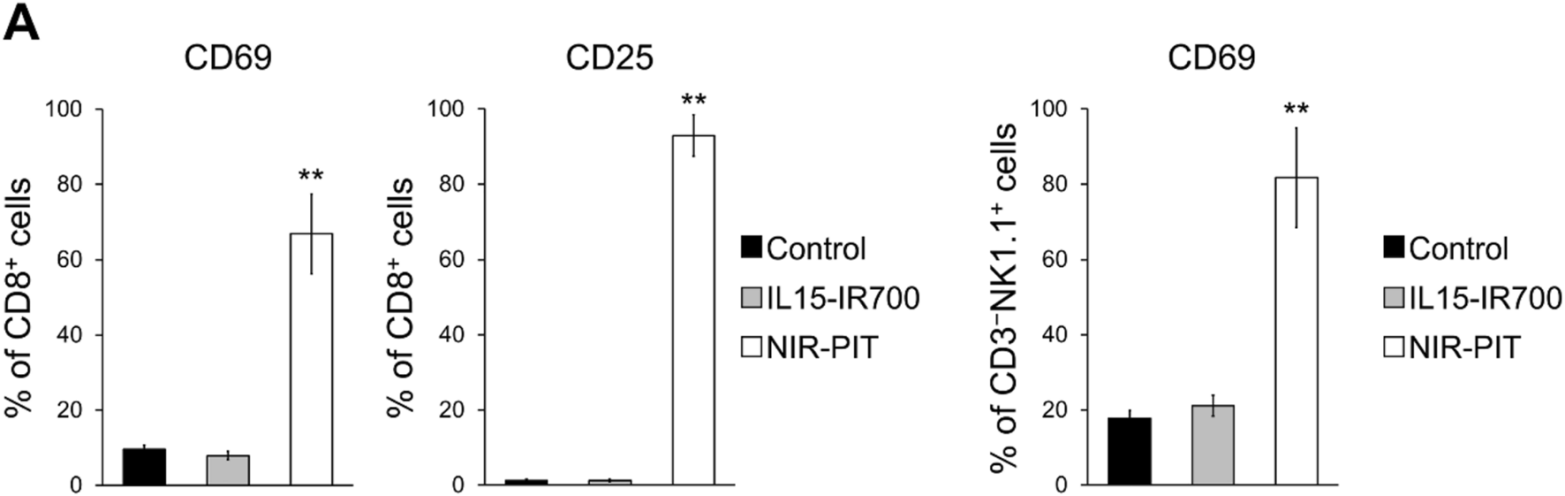
Phenotypic analysis of immune cells in the tumor-draining lymph node two days after NIR-PIT. The expression of CD69 and CD25 in CD8^+^ T cells **A** and natural killer cells **B** were analyzed by flow cytometry. Data are expressed as the mean ± SD. **P < 0.01 versus Control (n = 3–5, Tukey–Kramer test)

Next, the host immune response after cytokine-based NIR-PIT was assessed. Two days after NIR-PIT, lymphocyte activation markers, CD69 and CD25, were significantly elevated in CD8^+^ T cells and NK cells in TDLN (Fig. 6A and B). These results suggested that cytokine-based NIR-PIT could elicit cytotoxic T cells and NK cells activation in TDLN.

## Discussion

To our knowledge, this is the first application of a cytokine as the targeting moiety in an NIR-PIT agent. Most pre-clinical studies of NIR-PIT utilize antibody-based agents, and the only approved NIR-PIT agent is cetuximab-IR700. The availability of targeting molecules other than antibodies would expand treatment options and make NIR-PIT applicable to more carcinomas. In addition, cytokine-based NIR-PIT agents clear more rapidly than antibody-based agents avoiding associated skin phototoxicity [18]. Patients receiving antibody-based IR700 conjugates are advised to avoid exposure of skin and eyes to direct sunlight or bright indoor light for at least 4 weeks after completing treatment [19]. Since the molecular weight of cytokines is much smaller than antibodies, they have a shorter serum half-life and more rapid clearance [20]. Therefore, cytokine-based NIR-PIT may help patients return to normal life more quickly.

The routes of administration of therapeutic agents can affect the treatment efficacy. In most antibody-based therapies, including NIR-PIT, antibody agents are generally administered intravenously. Therefore, an intravenous injection was also attempted for cytokine-based therapy in this study. However, IL15-IR700 accumulation in the tumor was low, and little tissue changes were observed after light irradiation (Supplemental Figs. 4 and 5). By contrast, after intratumoral administration, IL15-IR700 remained in the tumor for at least 5 h, and significant therapeutic effects were induced by light irradiation (Figs. 2, 3 and 4). In cytokine-based immunotherapy, systemic therapy via intravenous or subcutaneous administration often results in limited therapeutic efficacy or causes severe adverse events, and therefore have not proven valid as strategies [11]. To overcome this problem, many clinical trials have used intratumoral administration of cytokines [21]. Although further validation is needed to determine the appropriate route of administration in cytokine-based NIR-PIT, intratumoral administration may be a promising therapeutic option.

In the current study, IL15 and IL15Rα were employed as carriers and targets of NIR-PIT agents respectively. The receptor of IL15 consists of the IL2 receptor β and γc subunits and IL15Rα. IL15 has higher affinity binding for IL15Rα (Kd = 10–80 pM) than IL2Rβ/γc (Kd = 0.27–2.5 nM) [22, 23]. This affinity is approximately 100-times higher than that of cetuximab-IR700 when binding its target, EGFR (Kd = 0.38 nM) [24]. The cytotoxic effects of NIR-PIT are induced by photochemical reactions on the cellular membrane; thus, carriers require robust binding to the receptor on the cellular membrane. It is unclear what affinity level of a monovalent cytokine is required for NIR-PIT. However, any cytokine with affinity equivalent or superior to cetuximab could be a potential candidate. In addition, while cytokine receptors are generally expressed on immune cells they tend to be overexpressed on various solid cancers. The overexpression of IL15Rα has been reported in head and neck cancer and triple-negative breast cancer [17, 25]. Indeed, significant cytotoxic effects were observed in human breast cancer MDA-MB-231 cells (Supplemental Fig. 3). Moreover, IL4, IL6, IL8, and IL21 receptors are overexpressed on the surface of various cancer cells, including bladder, ovarian, breast, colon, head and neck, lung, and pancreatic cancer [26–33]. Thus, cytokine-based NIR-PIT can be applied to treat a variety of cancer types and may be a novel therapeutic strategy.

Theoretically, host immune activation induced by IL15-based NIR-PIT combines with the biological effects of IL15. The rupture of cellular membranes by antibody-based NIR-PIT causes leakage of cellular contents and stimulates the host anti-cancer immune response [2]. In the current study, IL15-based NIR-PIT also induced morphological changes associated with membrane damage in vitro (Fig. 1C) and the increase of HSP70 and calreticulin suggested cytokine-based NIR-PIT induced ICD (Fig. 5). Moreover, the elevation of immune cell activation markers, CD69 and CD25, in CD8^+^ and NK cells was observed in TDLNs after NIR-PIT, indicating an immune activation (Fig. 6). Notably, the elevated level of these markers was dramatically higher than those observed in our previous antibody-based NIR-PIT study [13, 34]. This may be an augmentation effect caused by the use of IL15, one of the most promising cytokines to activate and expand CD8^+^ T and NK cells for cancer immunotherapy [35]. Various monotherapies using IL15 or its agonist and combination therapies in which IL15 is used along with other immunotherapies have been developed, and several clinical trials are ongoing [36]. In another study intratumoral IL15 injection improved the therapeutic efficacy of CD44-targeted NIR-PIT by activating CD8^+^ T and NK cells [12, 13]. Accordingly, in the current study, the therapeutic effects of NIR-PIT using IL15-IR700 might be potentiated by the immunostimulatory effects of IL15-IR700 itself. Immunostimulatory cytokine-based NIR-PIT could be a new treatment option because the NIR-PIT itself is expected to have an immune-potentiating effect.

This study has several limitations. In subcutaneous models, intratumoral administration is relatively easy. However, in the clinical setting, the agents often require image guidance to better identify the tumor location, adding the complexity of the procedure. Additionally, sophisticated techniques are needed to ensure uniform distribution of the agents within the tumor to avoid “dead zones” where the agent is not dispersed. Moreover, we only used a single concentration of the IL15 NIR-PIT agent. Further studies are needed to optimize the injection dose, volume, site, and speed, which may affect therapeutic outcomes [37].

In conclusion, IL15-based NIR-PIT showed cytotoxic effects in vitro, and suppressed tumor proliferation, improved survival, and elicited host immune response in vivo. In addition, intratumoral administration of NIR-PIT agents showed significant therapeutic effects. These results indicate that cytokine-based NIR-PIT may be a new treatment option for some tumors.

## Supplementary Information

Below is the link to the electronic supplementary material.

Supplementary file 1 (PDF 484 KB)

## Data Availability

The data presented in this study is available on request from the corresponding author.

## Abbreviations

DIC: Differential interference contrast
DIG: Digoxigenin
EGFR: Epidermal growth factor receptor
HSP70: Heatshock protein 70
ICD: Immunogenic cell death
IL15: Interleukin-15
NIR-PIT: Near-infrared photoimmunotherapy
