# Supplementary material for: Interleukin 15 (IL15)-based near-infrared photoimmunotherapy
Source: Cancer Immunol Immunother. 2025 Aug 31;74(9):295. doi: 10.1007/s00262-025-04151-8 (PMC12399473; doi:10.1007/s00262-025-04151-8)
Supplement: Supplementary file 1 — (PDF 484 KB) [file 262_2025_4151_MOESM1_ESM.pdf]

Supplementary Information

Interleukin 15 (IL15)-based near-infrared photoimmunotherapy

Motofumi Suzuki, Aki Furusawa, Hiroshi Yamamoto, Makoto Kano, Miyu Kano, Seiichiro Takao, Shuhei Okuyama, Peter L. Choyke, and Hisataka Kobayashi

Molecular Imaging Branch, Center for Cancer Research, National Cancer Institute, National Institutes of Health, Bethesda, MD, 20892, USA

Supplemental figure 1

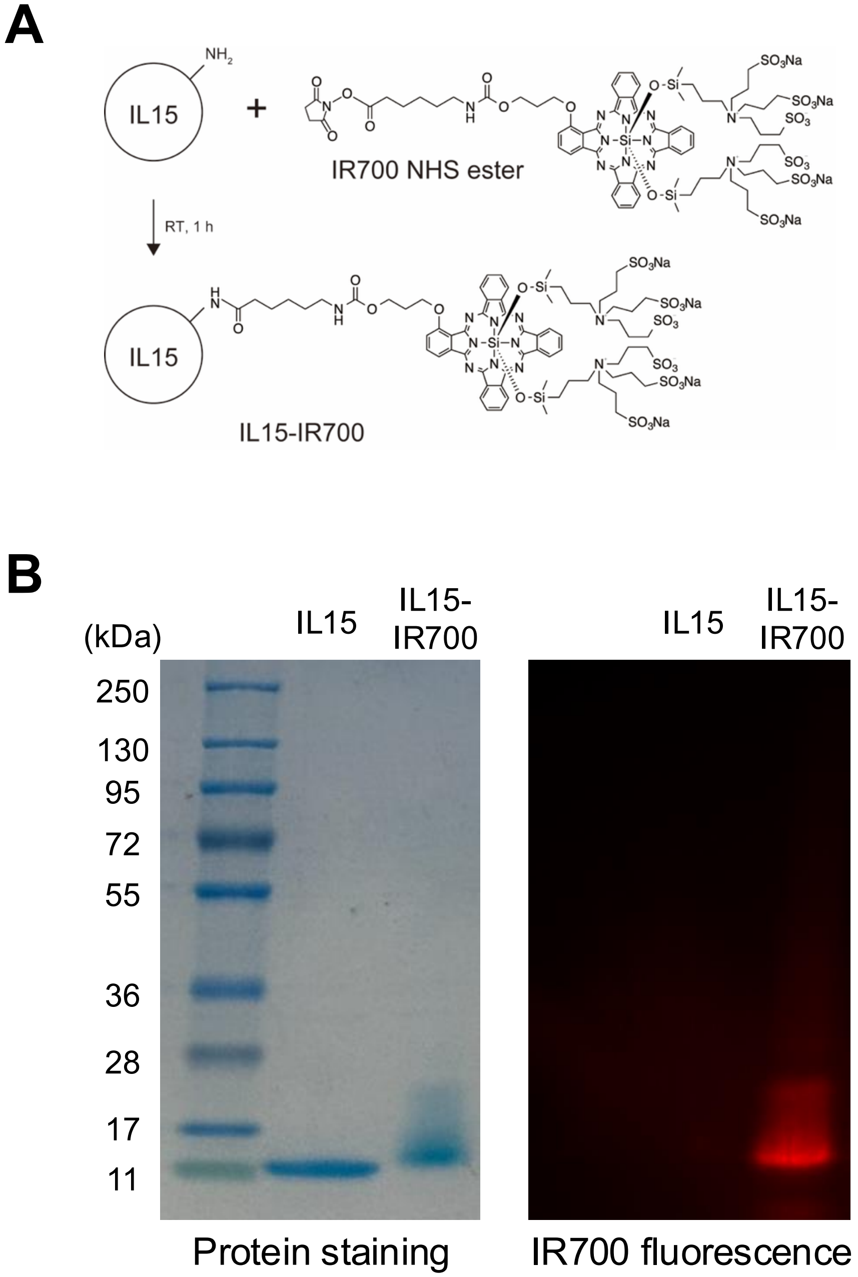

Supplemental figure 1
(A) Conjugation scheme of IL15-IR700. (B) Validation of IL15-IR700 by SDS-PAGE (left: protein staining, right; fluorescence at 700 nm).

Supplemental figure 2

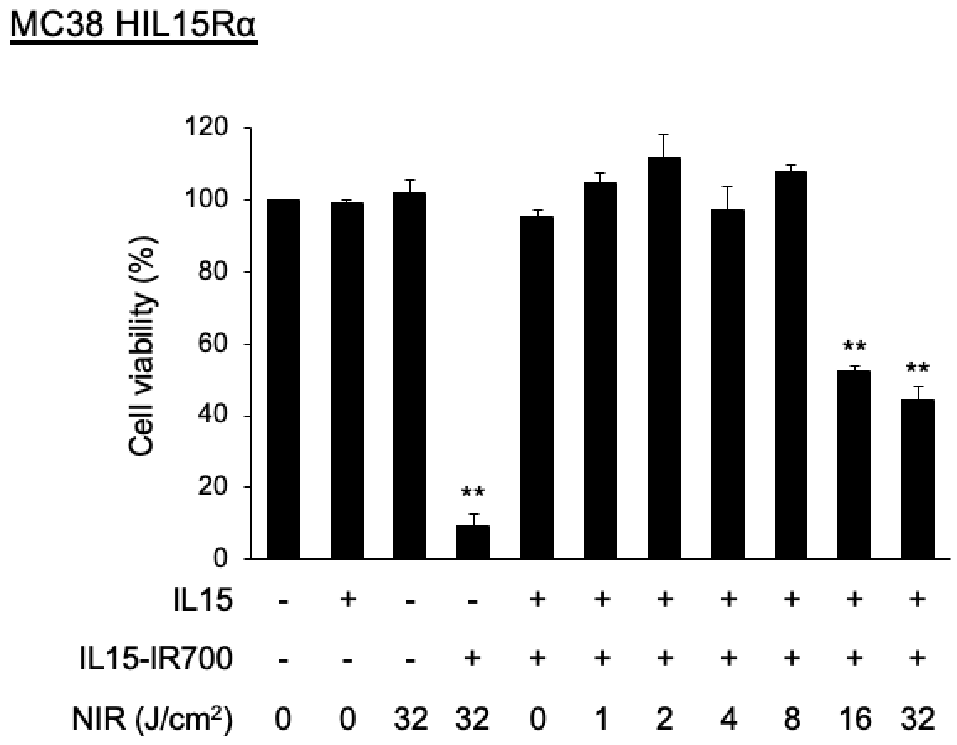

Supplemental figure 2
Selectivity of NIR-PIT using IL15-IR700. Cell viability after NIR-PIT in MC38 HIL15Rα cells after blocking with non-conjugated IL15. Data are expressed as the mean ± SD of three independent experiments. ** $P < 0.01$ versus Control (Student's t -test).

Supplemental figure 3

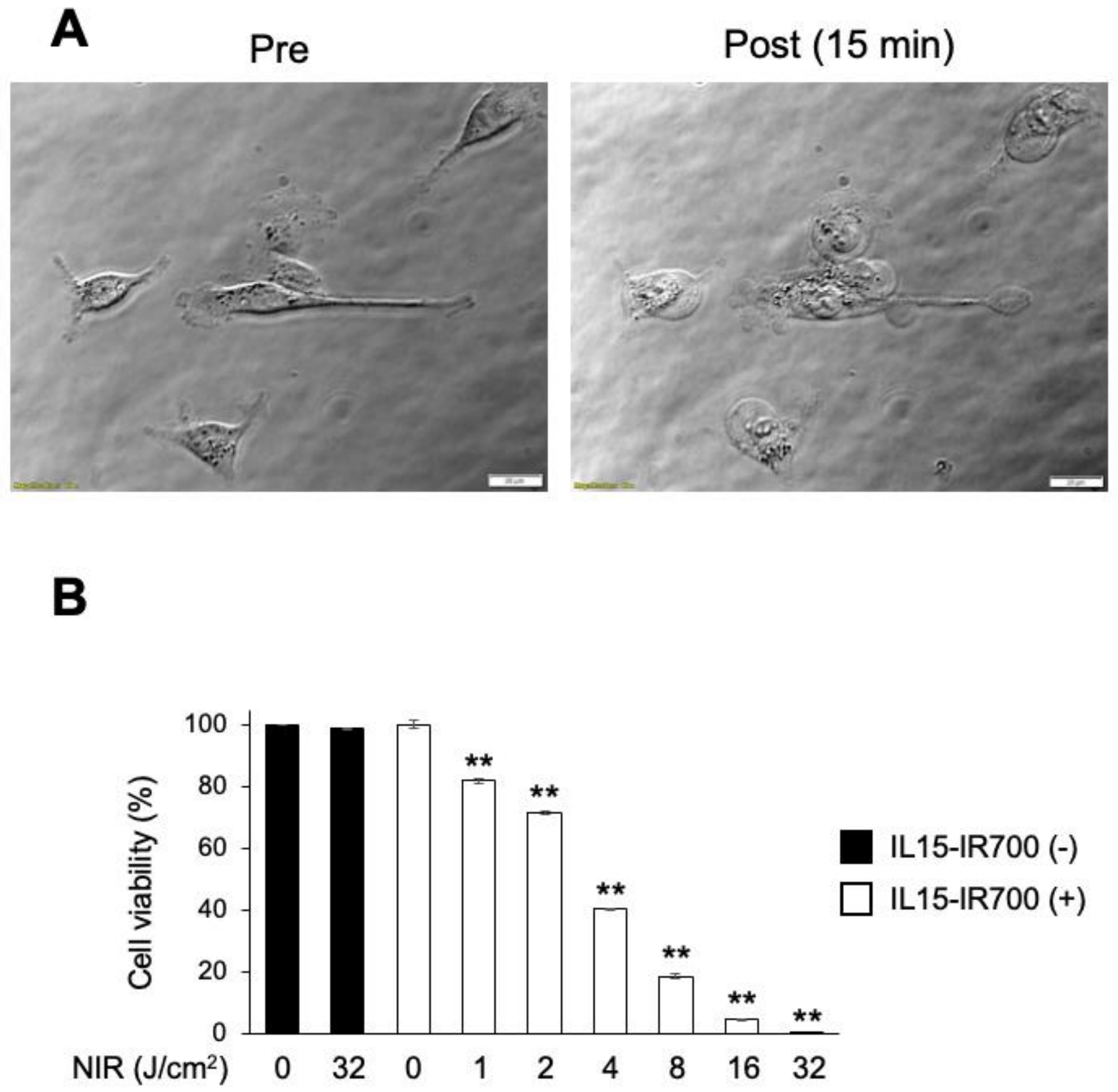

Supplemental figure 3

Cytotoxic effects of NIR-PIT using IL15-IR700 in human cancer cells. (A) Representative images of morphological changes after NIR-PIT. Scale bar, 20 µm. Pre, before irradiation; Post, after irradiation. (B) Cell viability after NIR-PIT in MDA-MB-231 cells was evaluated by MTT assay. Data are expressed as the mean ± SD of three independent experiments. ** $P < 0.01$ versus Control (Student's *t* -test).

Supplemental figure 4

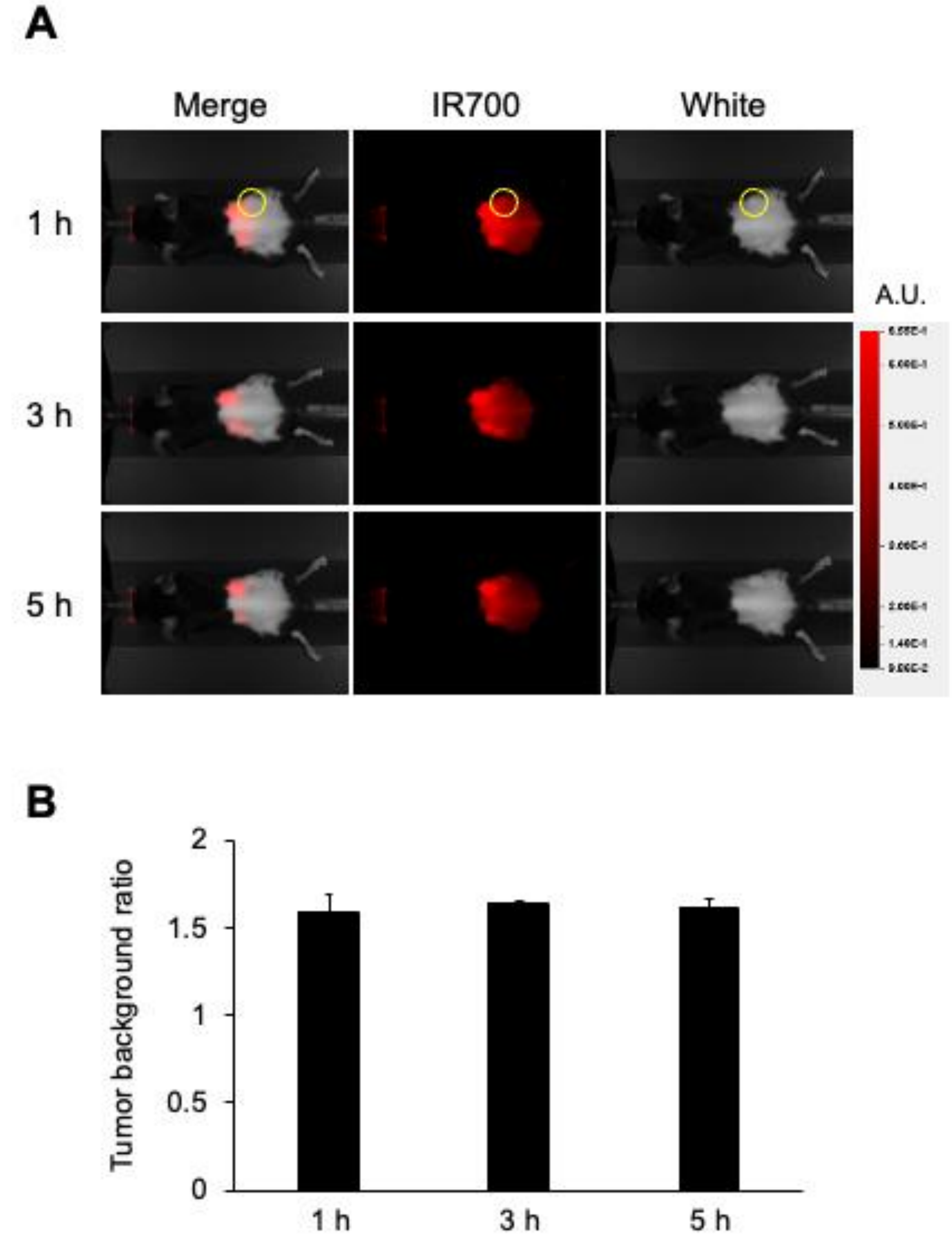

Supplemental figure 4

Accumulation of IL15-IR700 in tumor tissue. (A) Fluorescence images taken after intravenous injection of IL15-IR700. (B) Quantitative analysis of tumor-background fluorescence intensity ratio. Data are expressed as the mean ± SD.

Supplemental figure 5

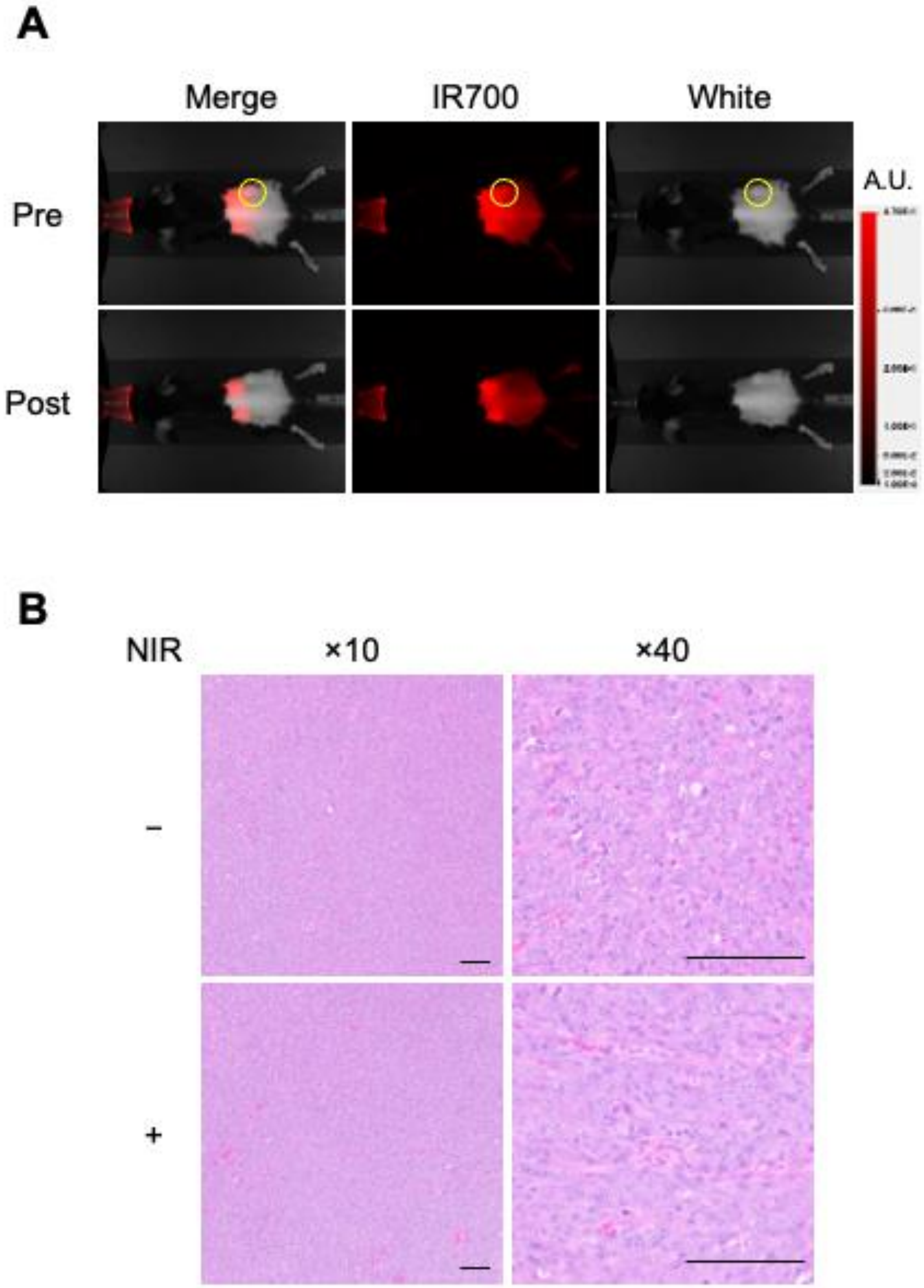

Supplemental figure 5

In vivo NIR-PIT in an MC38 HIL15Rα tumor-bearing mouse model with intravenously injected IL15-IR700. (A) Fluorescence images taken before and immediately after NIR-PIT on Days 7. (B) Representative HE-staining of the tumor sections. Scale bar, 100 μm.

Supplementary methods

Validation of IL15-IR700

The conjugation of IR700 to IL15 was assessed by sodium dodecyl sulfate–polyacrylamide gel electrophoresis using a 4–12% gradient polyacrylamide gel (Life Technologies). Unconjugated IL15 was used as a control. The fluorescence image was obtained using Pearl Imager (LI-COR Biosciences). The gel was then stained with Colloidal Blue to compare the molecular weight of IL15 with or without IR700.
